# 4-Chloro-5-(di­methyl­amino)-2-[(5-phenyl-1,3,4-oxa­diazol-2-yl)meth­yl]pyridazin-3(2*H*)-one

**DOI:** 10.1107/S241431462200342X

**Published:** 2022-03-31

**Authors:** Jingjing Song, Xinyu Jiang, Ziyi Wang, Jingyao Pei, Hongsen Li

**Affiliations:** a College of Chemistry and Chemical Engineering, Shanghai University of Engineering Science, 333 Longteng Road, Shanghai, People’s Republic of China; Katholieke Universiteit Leuven, Belgium

**Keywords:** crystal structure, pyridazinone, oxa­diazole, hydrogen bonding

## Abstract

In the structure of the title compound, C_15_H_14_ClN_5_O_2_, the terminal phenyl ring and the adjacent furan ring subtend a dihedral angle of 6.77 (17)°. The 4-chloro-5-(di­methyl­amino)-pyridazin-3(2*H*)-one group is linked to the oxa­diazole ring by a methyl­ene bridge, and the dihedral angle between the pyridazine and oxa­diazole rings is 88.66 (14)°. In the crystal, C—H⋯O and C—H⋯N hydrogen bonds extend the structure into a three-dimensional network.

## Abstract 2

The title compound crystallizes in the monoclinic space group *P*2_1_. The crystal packing is characterized by C—H⋯N and C—H⋯O contacts.

## Graphical abstract

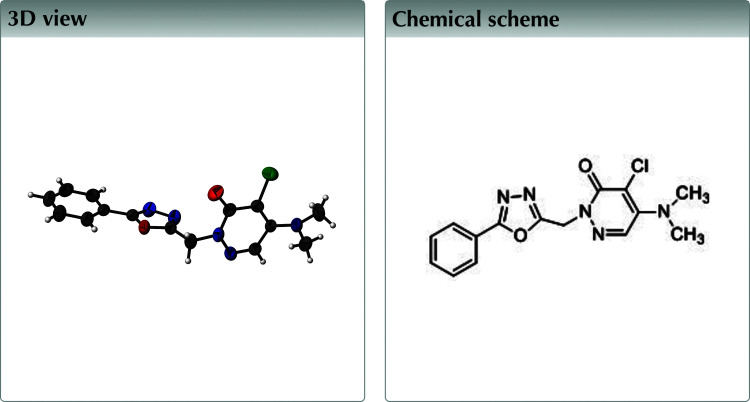

## Structure description

Pyridazinones have attracted increasing attention as a scaffold because of their wide spectrum of biological activity (Zou *et al.*, 2002 ▸). Along with the development of design and synthetic methodology, pyridazinone derivatives have been widely applied in medicinal and agricultural chemistry (Arora *et al.*, 2022 ▸; Vaidergorn *et al.*, 2021 ▸; Zhang *et al.*, 2020 ▸; Lu *et al.*, 2017 ▸; Cao *et al.*, 2005 ▸; Xu *et al.*, 2008 ▸; Sun *et al.*, 2015 ▸). As part of our work in this area, a series of pyridazinone derivatives containing an oxa­diazole moiety have been designed and synthesized, and we report here the crystal structure of the tittle compound.

**Figure 1:**
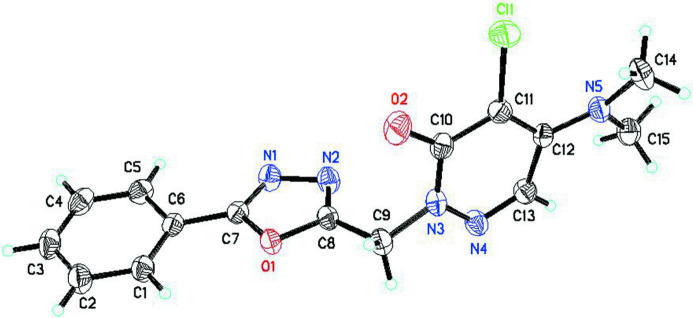
The mol­ecular structure of the title compound, with the atom labelling and displacement ellipsoids drawn at the 50% probability level.

The mol­ecular structure of the title compound is shown in Fig. 1 ▸. The phenyl (C1–C6) and oxa­diazole (O1/N1/N2/C7/C8) rings are almost coplanar, subtending a dihedral angle of 6.77 (17)°. The pyridazine ring is almost perpendicular to oxa­diazole ring, making a dihedral angle of 88.66 (14)°. The dihedral angle between the phenyl and pyridazine rings is 82.01 (17)°.

**Figure 2:**
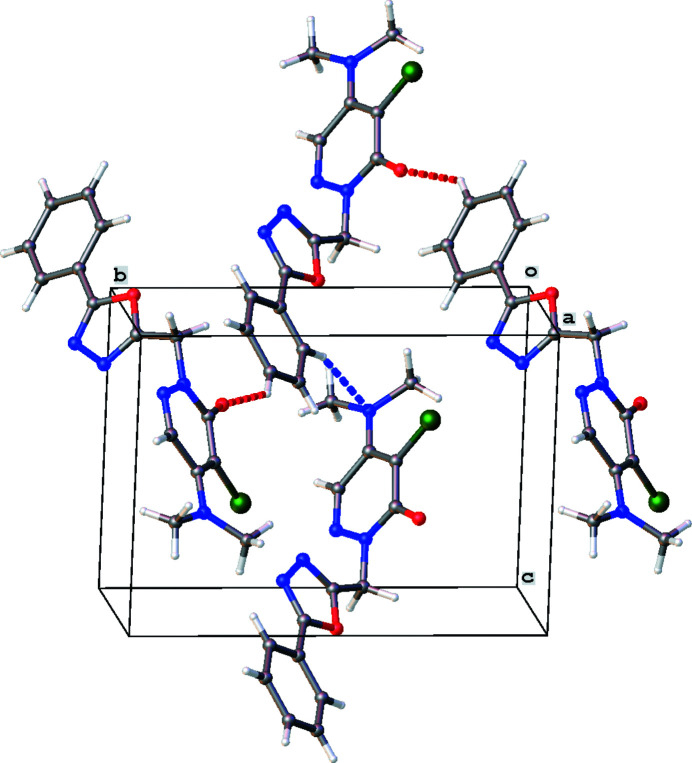
The crystal packing of the title compound. The C—H⋯N and C—H⋯O hydrogen bonds are shown as dashed lines (see also Table 1 ▸).

The crystal packing is characterized by C—H⋯N and C—H⋯O contacts (Fig. 2 ▸, Table 1 ▸).

## Synthesis and crystallization

To a 100 ml round-bottom flask, 4,5-di­chloro-2-[(5-phenyl-1,3,4-oxa­diazol-2-yl) meth­yl]pyridazin-3(2*H*)-one (1.0 g, 3.1 mmol), di­methyl­amine (0.6 ml, 6.2 mmol), and potassium carbonate (0.86 g, 6.2 mmol) were added in 30 ml of DMF and stirred for 8 h at 353 K. Afterwards, the reaction mixture was cooled and poured into 60 ml of ice–water. The precipitate formed was collected by filtration and then dried to obtain the pure title compound (yield 0.56 g, 54.6%). It was recrystallized from mixed solvents of ethyl acetate and petroleum (3:5) to give crystals suitable for X-ray diffraction (m.p. 427–429 K).

^1^H NMR (400 MHz, CDCl_3_): 3.18 (*s*, 6H), 5.62 (*s*, 2H), 7.54 (*m*, 3H), 7.67 (*s*, 1*H*), 8.06 (*dd*, 2H, *J* = 7.6 Hz, *J* = 1.6 Hz). IR (KBr, cm^−1^): 2973, 2937, 1632, 1600, 1520, 1482, 1449, 1216, 1146, 752, 710.

## Refinement

Crystal data, data collection and structure refinement details are summarized in Table 2 ▸.

## Supplementary Material

Crystal structure: contains datablock(s) I. DOI: 10.1107/S241431462200342X/vm4052sup1.cif

Structure factors: contains datablock(s) I. DOI: 10.1107/S241431462200342X/vm4052Isup2.hkl

Supporting information file. DOI: 10.1107/S241431462200342X/vm4052Isup3.cml

CCDC reference: 2162169

Additional supporting information:  crystallographic information; 3D view; checkCIF report

## Figures and Tables

**Table 1:** Hydrogen-bond geometry (Å, °)

*D*—H⋯*A*	*D*—H	H⋯*A*	*D*⋯*A*	*D*—H⋯*A*
C3—H3⋯O2^i^	0.93	2.36	3.125 (5)	140
C1—H1⋯N5^ii^	0.93	2.61	3.477 (5)	156

Symmetry codes: (i) 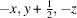 ; (ii) 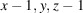 .

**Table 2:** Experimental details

Crystal data	
Chemical formula	C_15_H_14_ClN_5_O_2_
*M*_r_	331.76
Crystal system, space group	Monoclinic, *P*2_1_
Temperature (K)	293
*a*, *b*, *c* (Å)	7.1533 (16), 11.936 (3), 9.004 (2)
β (°)	98.128 (5)
*V* (Å^3^)	761.1 (3)
*Z*	2
Radiation type	Mo *K*α
μ (mm^−1^)	0.27
Crystal size (mm)	0.22 × 0.17 × 0.12

Data collection	
Diffractometer	Bruker *SMART* CCD area detector
Absorption correction	Multi-scan (*SADABS*; Bruker, 2002 ▸)
*T*_min_, *T*_max_	0.580, 0.746
No. of measured, independent and observed [*I* > 2σ(*I*)] reflections	4542, 2915, 2673
*R*_int_	0.030
(sin θ/λ)_max_ (Å^−1^)	0.617

Refinement	
*R*[*F* ^2^ > 2σ(*F* ^2^)], *wR*(*F* ^2^), *S*	0.042, 0.100, 1.04
No. of reflections	2915
No. of parameters	211
No. of restraints	1
H-atom treatment	H-atom parameters constrained
Δρ_max_, Δρ_min_ (e Å^−3^)	0.28, −0.30
Absolute structure	Flack *x* determined using 1139 quotients [(*I* ^+^)−(*I* ^−^)]/[(*I* ^+^)+(*I* ^−^)] (Parsons *et al.*, 2013 ▸)
Absolute structure parameter	0.00 (4)

Computer programs: *SMART* and *SAINT* (Bruker, 2002 ▸), *SHELXTL* (Sheldrick, 2008 ▸) and *SHELXL2013* (Sheldrick, 2015 ▸).

## full crystallographic data

### Crystal data

**Table:**

C_15_H_14_ClN_5_O_2_	*F*(000) = 344
*M**_r_* = 331.76	*D*_x_ = 1.448 Mg m^−^^3^
Monoclinic, *P*2_1_	Mo *K*α radiation, λ = 0.71073 Å
*a* = 7.1533 (16) Å	Cell parameters from 2107 reflections
*b* = 11.936 (3) Å	θ = 5.7–49.7°
*c* = 9.004 (2) Å	µ = 0.27 mm^−^^1^
β = 98.128 (5)°	*T* = 293 K
*V* = 761.1 (3) Å^3^	Prismatic, colorless
*Z* = 2	0.22 × 0.17 × 0.12 mm

### Data collection

**Table:**

Bruker SMART CCD area detector diffractometer	2673 reflections with *I* > 2σ(*I*)
phi and ω scans	*R*_int_ = 0.030
Absorption correction: multi-scan (SADABS; Bruker, 2002)	θ_max_ = 26.0°, θ_min_ = 2.3°
*T*_min_ = 0.580, *T*_max_ = 0.746	*h* = −8→8
4542 measured reflections	*k* = −14→14
2915 independent reflections	*l* = −9→11

### Refinement

**Table:**

Refinement on *F*^2^	H-atom parameters constrained
Least-squares matrix: full	*w* = 1/[σ^2^(*F*_o_^2^) + (0.0624*P*)^2^] where *P* = (*F*_o_^2^ + 2*F*_c_^2^)/3
*R*[*F*^2^ > 2σ(*F*^2^)] = 0.042	(Δ/σ)_max_ < 0.001
*wR*(*F*^2^) = 0.100	Δρ_max_ = 0.28 e Å^−^^3^
*S* = 1.04	Δρ_min_ = −0.30 e Å^−^^3^
2915 reflections	Extinction correction: SHELXL, Fc^*^=kFc[1+0.001xFc^2^λ^3^/sin(2θ)]^-1/4^
211 parameters	Extinction coefficient: 0.029 (8)
1 restraint	Absolute structure: Flack *x* determined using 1139 quotients [(*I*^+^)-(*I*^-^)]/[(*I*^+^)+(*I*^-^)] (Parsons *et al.*, 2013)
Hydrogen site location: inferred from neighbouring sites	Absolute structure parameter: 0.00 (4)

### Special details

**Table:**

Geometry. All esds (except the esd in the dihedral angle between two l.s. planes) are estimated using the full covariance matrix. The cell esds are taken into account individually in the estimation of esds in distances, angles and torsion angles; correlations between esds in cell parameters are only used when they are defined by crystal symmetry. An approximate (isotropic) treatment of cell esds is used for estimating esds involving l.s. planes.

### Fractional atomic coordinates and isotropic or equivalent isotropic displacement parameters (Å^2^)

**Table:**

	*x*	*y*	*z*	*U*_iso_*/*U*_eq_	
Cl1	0.85200 (15)	0.73449 (8)	0.58094 (11)	0.0698 (3)	
N1	0.3672 (4)	1.1087 (2)	0.1248 (3)	0.0522 (7)	
N2	0.5386 (4)	1.0502 (2)	0.1672 (3)	0.0525 (7)	
N3	0.8278 (4)	0.8804 (2)	0.1888 (3)	0.0450 (6)	
N4	0.9890 (4)	0.9391 (2)	0.1928 (3)	0.0503 (7)	
N5	1.2442 (4)	0.8512 (2)	0.5493 (3)	0.0501 (7)	
O1	0.3908 (3)	0.97490 (18)	−0.0393 (2)	0.0446 (5)	
O2	0.6230 (4)	0.7702 (2)	0.2898 (3)	0.0695 (8)	
C1	0.0415 (5)	1.0303 (3)	−0.2139 (4)	0.0530 (8)	
H1	0.1196	0.9776	−0.2501	0.064*	
C2	−0.1339 (5)	1.0519 (4)	−0.2928 (4)	0.0629 (10)	
H2	−0.1736	1.0139	−0.3818	0.075*	
C3	−0.2509 (5)	1.1296 (3)	−0.2406 (5)	0.0619 (10)	
H3	−0.3693	1.1446	−0.2939	0.074*	
C4	−0.1906 (6)	1.1849 (3)	−0.1086 (5)	0.0605 (9)	
H4	−0.2695	1.2372	−0.0726	0.073*	
C5	−0.0155 (5)	1.1641 (3)	−0.0288 (4)	0.0526 (8)	
H5	0.0237	1.2023	0.0601	0.063*	
C6	0.1024 (4)	1.0857 (3)	−0.0819 (3)	0.0425 (7)	
C7	0.2862 (4)	1.0612 (2)	0.0054 (3)	0.0406 (7)	
C8	0.5443 (4)	0.9750 (3)	0.0679 (3)	0.0422 (7)	
C9	0.6961 (5)	0.8919 (3)	0.0516 (4)	0.0502 (8)	
H9A	0.6391	0.8196	0.0245	0.060*	
H9B	0.7642	0.9154	−0.0289	0.060*	
C10	0.7789 (4)	0.8150 (3)	0.3034 (4)	0.0453 (7)	
C11	0.9230 (5)	0.8076 (3)	0.4331 (3)	0.0449 (7)	
C12	1.0946 (4)	0.8589 (3)	0.4373 (3)	0.0396 (6)	
C13	1.1132 (5)	0.9282 (3)	0.3105 (4)	0.0473 (8)	
H13	1.2245	0.9690	0.3132	0.057*	
C14	1.2910 (6)	0.7498 (4)	0.6345 (4)	0.0692 (11)	
H14A	1.2811	0.7631	0.7382	0.104*	
H14B	1.4178	0.7277	0.6249	0.104*	
H14C	1.2052	0.6912	0.5968	0.104*	
C15	1.4001 (5)	0.9309 (4)	0.5556 (4)	0.0636 (10)	
H15A	1.4852	0.9073	0.4882	0.095*	
H15B	1.4664	0.9339	0.6559	0.095*	
H15C	1.3513	1.0038	0.5266	0.095*	

### Atomic displacement parameters (Å^2^)

**Table:**

	*U*^11^	*U*^22^	*U*^33^	*U*^12^	*U*^13^	*U*^23^
Cl1	0.0676 (6)	0.0740 (6)	0.0695 (6)	−0.0110 (5)	0.0159 (4)	0.0263 (5)
N1	0.0468 (16)	0.0478 (15)	0.0600 (17)	0.0006 (13)	0.0010 (13)	−0.0103 (13)
N2	0.0476 (16)	0.0503 (15)	0.0563 (15)	−0.0009 (13)	−0.0047 (12)	−0.0085 (14)
N3	0.0368 (14)	0.0436 (14)	0.0518 (14)	0.0001 (11)	−0.0033 (11)	0.0016 (12)
N4	0.0466 (15)	0.0496 (15)	0.0525 (15)	−0.0048 (12)	−0.0002 (13)	0.0126 (12)
N5	0.0424 (15)	0.0559 (15)	0.0498 (15)	0.0020 (12)	−0.0017 (12)	0.0040 (12)
O1	0.0411 (12)	0.0456 (11)	0.0444 (11)	0.0056 (10)	−0.0034 (9)	−0.0044 (9)
O2	0.0435 (14)	0.0800 (19)	0.0831 (17)	−0.0224 (13)	0.0021 (12)	0.0027 (14)
C1	0.0453 (19)	0.057 (2)	0.0552 (19)	0.0100 (16)	0.0037 (15)	−0.0011 (16)
C2	0.050 (2)	0.070 (2)	0.066 (2)	0.0062 (18)	−0.0032 (17)	0.0008 (19)
C3	0.0411 (19)	0.063 (2)	0.079 (3)	0.0094 (17)	0.0004 (17)	0.0143 (19)
C4	0.051 (2)	0.0516 (19)	0.083 (3)	0.0150 (17)	0.0229 (18)	0.0122 (18)
C5	0.054 (2)	0.0462 (18)	0.0600 (19)	0.0089 (15)	0.0150 (16)	0.0052 (14)
C6	0.0381 (16)	0.0404 (15)	0.0499 (16)	0.0022 (13)	0.0093 (13)	0.0089 (12)
C7	0.0404 (15)	0.0377 (15)	0.0444 (15)	0.0012 (13)	0.0085 (13)	0.0039 (13)
C8	0.0393 (16)	0.0423 (15)	0.0428 (15)	−0.0037 (13)	−0.0015 (12)	−0.0004 (13)
C9	0.0467 (18)	0.0508 (19)	0.0503 (17)	0.0055 (15)	−0.0028 (14)	−0.0043 (15)
C10	0.0389 (17)	0.0411 (15)	0.0557 (18)	−0.0027 (13)	0.0060 (14)	0.0000 (14)
C11	0.0470 (18)	0.0407 (15)	0.0478 (16)	0.0018 (14)	0.0099 (14)	0.0076 (13)
C12	0.0362 (15)	0.0386 (14)	0.0433 (15)	0.0041 (12)	0.0034 (12)	−0.0002 (12)
C13	0.0387 (17)	0.0497 (17)	0.0526 (18)	−0.0100 (14)	0.0033 (15)	0.0062 (15)
C14	0.068 (3)	0.071 (2)	0.064 (2)	0.015 (2)	−0.0101 (18)	0.013 (2)
C15	0.0431 (19)	0.085 (3)	0.060 (2)	−0.0084 (19)	−0.0026 (16)	−0.0018 (19)

### Geometric parameters (Å, º)

**Table:**

Cl1—C11	1.727 (3)	C3—H3	0.9300
N1—C7	1.279 (4)	C4—C5	1.376 (6)
N1—N2	1.415 (4)	C4—H4	0.9300
N2—C8	1.272 (4)	C5—C6	1.389 (5)
N3—N4	1.345 (4)	C5—H5	0.9300
N3—C10	1.378 (4)	C6—C7	1.462 (4)
N3—C9	1.450 (4)	C8—C9	1.494 (5)
N4—C13	1.290 (4)	C9—H9A	0.9700
N5—C12	1.366 (4)	C9—H9B	0.9700
N5—C14	1.447 (5)	C10—C11	1.447 (4)
N5—C15	1.461 (5)	C11—C12	1.367 (5)
O1—C8	1.355 (3)	C12—C13	1.432 (4)
O1—C7	1.367 (4)	C13—H13	0.9300
O2—C10	1.227 (4)	C14—H14A	0.9600
C1—C6	1.376 (5)	C14—H14B	0.9600
C1—C2	1.376 (5)	C14—H14C	0.9600
C1—H1	0.9300	C15—H15A	0.9600
C2—C3	1.376 (6)	C15—H15B	0.9600
C2—H2	0.9300	C15—H15C	0.9600
C3—C4	1.374 (6)		
			
C7—N1—N2	106.3 (3)	N2—C8—C9	129.4 (3)
C8—N2—N1	105.7 (2)	O1—C8—C9	117.2 (3)
N4—N3—C10	125.6 (3)	N3—C9—C8	111.9 (3)
N4—N3—C9	115.2 (3)	N3—C9—H9A	109.2
C10—N3—C9	119.2 (3)	C8—C9—H9A	109.2
C13—N4—N3	117.3 (3)	N3—C9—H9B	109.2
C12—N5—C14	123.1 (3)	C8—C9—H9B	109.2
C12—N5—C15	119.9 (3)	H9A—C9—H9B	107.9
C14—N5—C15	114.0 (3)	O2—C10—N3	119.7 (3)
C8—O1—C7	102.2 (2)	O2—C10—C11	126.0 (3)
C6—C1—C2	120.7 (3)	N3—C10—C11	114.3 (3)
C6—C1—H1	119.7	C12—C11—C10	122.0 (3)
C2—C1—H1	119.7	C12—C11—Cl1	124.4 (2)
C3—C2—C1	120.3 (4)	C10—C11—Cl1	113.5 (2)
C3—C2—H2	119.9	N5—C12—C11	126.6 (3)
C1—C2—H2	119.9	N5—C12—C13	118.4 (3)
C4—C3—C2	119.2 (4)	C11—C12—C13	114.9 (3)
C4—C3—H3	120.4	N4—C13—C12	125.6 (3)
C2—C3—H3	120.4	N4—C13—H13	117.2
C3—C4—C5	121.1 (3)	C12—C13—H13	117.2
C3—C4—H4	119.5	N5—C14—H14A	109.5
C5—C4—H4	119.5	N5—C14—H14B	109.5
C4—C5—C6	119.6 (4)	H14A—C14—H14B	109.5
C4—C5—H5	120.2	N5—C14—H14C	109.5
C6—C5—H5	120.2	H14A—C14—H14C	109.5
C1—C6—C5	119.2 (3)	H14B—C14—H14C	109.5
C1—C6—C7	121.3 (3)	N5—C15—H15A	109.5
C5—C6—C7	119.5 (3)	N5—C15—H15B	109.5
N1—C7—O1	112.3 (3)	H15A—C15—H15B	109.5
N1—C7—C6	129.0 (3)	N5—C15—H15C	109.5
O1—C7—C6	118.7 (3)	H15A—C15—H15C	109.5
N2—C8—O1	113.4 (3)	H15B—C15—H15C	109.5
			
C7—N1—N2—C8	1.0 (4)	N4—N3—C9—C8	−98.6 (4)
C10—N3—N4—C13	4.2 (5)	C10—N3—C9—C8	79.8 (4)
C9—N3—N4—C13	−177.5 (3)	N2—C8—C9—N3	16.7 (5)
C6—C1—C2—C3	0.0 (6)	O1—C8—C9—N3	−165.6 (3)
C1—C2—C3—C4	−0.2 (6)	N4—N3—C10—O2	175.9 (3)
C2—C3—C4—C5	0.3 (6)	C9—N3—C10—O2	−2.2 (4)
C3—C4—C5—C6	−0.2 (5)	N4—N3—C10—C11	−3.3 (4)
C2—C1—C6—C5	0.1 (5)	C9—N3—C10—C11	178.6 (3)
C2—C1—C6—C7	178.2 (3)	O2—C10—C11—C12	178.8 (3)
C4—C5—C6—C1	0.0 (5)	N3—C10—C11—C12	−2.0 (4)
C4—C5—C6—C7	−178.1 (3)	O2—C10—C11—Cl1	−4.0 (4)
N2—N1—C7—O1	−0.9 (4)	N3—C10—C11—Cl1	175.2 (2)
N2—N1—C7—C6	177.5 (3)	C14—N5—C12—C11	35.7 (5)
C8—O1—C7—N1	0.5 (3)	C15—N5—C12—C11	−165.2 (3)
C8—O1—C7—C6	−178.1 (3)	C14—N5—C12—C13	−145.3 (3)
C1—C6—C7—N1	176.0 (3)	C15—N5—C12—C13	13.8 (5)
C5—C6—C7—N1	−5.9 (5)	C10—C11—C12—N5	−175.4 (3)
C1—C6—C7—O1	−5.7 (4)	Cl1—C11—C12—N5	7.8 (5)
C5—C6—C7—O1	172.5 (3)	C10—C11—C12—C13	5.6 (4)
N1—N2—C8—O1	−0.7 (4)	Cl1—C11—C12—C13	−171.3 (2)
N1—N2—C8—C9	177.1 (3)	N3—N4—C13—C12	0.1 (5)
C7—O1—C8—N2	0.2 (4)	N5—C12—C13—N4	176.0 (3)
C7—O1—C8—C9	−177.9 (3)	C11—C12—C13—N4	−4.9 (5)

### Hydrogen-bond geometry (Å, º)

**Table:**

*D*—H···*A*	*D*—H	H···*A*	*D*···*A*	*D*—H···*A*
C14—H14*C*···Cl1	0.96	2.56	3.114 (4)	117
C1—H1···O1	0.93	2.52	2.836 (4)	100
C3—H3···O2^i^	0.93	2.36	3.125 (5)	140
C1—H1···N5^ii^	0.93	2.61	3.477 (5)	156

Symmetry codes: (i) −*x*, *y*+1/2, −*z*; (ii) *x*−1, *y*, *z*−1.
